# Chronic Eosinophilic Leukemia—Not Otherwise Specified (NOS) in the Background of a Large Cell Lymphoma

**DOI:** 10.1155/2013/458303

**Published:** 2013-10-09

**Authors:** Wilson I. Gonsalves, Rong He, Animesh Pardanani, Vinay Gupta, Jacob P. Smeltzer, Curtis A. Hanson, Thomas E. Witzig

**Affiliations:** ^1^Division of Hematology, Department of Medicine, Mayo Clinic, First Street SW, Rochester, MN 55905, USA; ^2^Division of Hematopathology, Department of Laboratory Medicine and Pathology, Rochester, MN 55905, USA

## Abstract

Clonal eosinophilic disorders are rare among hematological malignancies. Most eosinophilia tends to be due to secondary causes such as infections, hypersensitivity conditions, drug reactions, and connective tissue disorders. The presence of a primary clonal eosinophilic disorder such as chronic eosinophilic leukemia—not otherwise specified (NOS) in the presence of a synchronous large cell lymphoma—is rare making the diagnosis challenging. We present a case of a 51-year-old female with the aforementioned presentation and demonstrate the extensive workup performed to identify the diagnosis.

## 1. Introduction

Non-Hodgkin's lymphoma (NHL) is the most common hematological malignancy in the United States [1]; in contrast, clonal eosinophilic disorders are rare [2]. Lymphomas, particularly T-cell [3] and Hodgkin's [4], can be associated with a reactive eosinophilia due to the production of various cytokines that promote eosinophil differentiation and survival. We report a rare case of a patient presenting with chronic eosinophilic leukemia in the setting of a CD30^+^ large cell lymphoma.

## 2. Case Report

**Figure 1:**
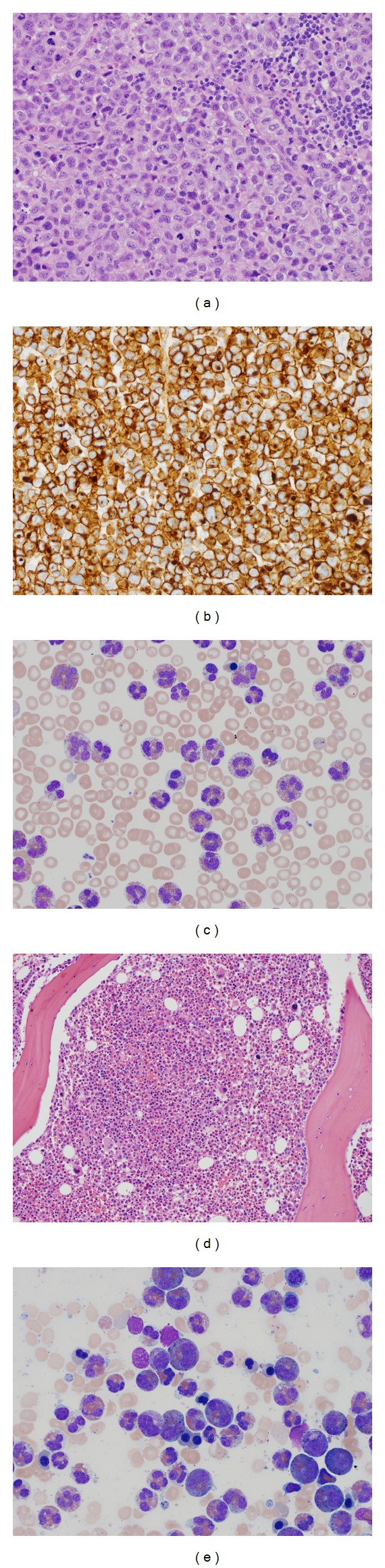
(a) Excisional biopsy of cervical lymph node showing sheets of large atypical lymphoid cells with frequent mitoses. (b) Positive CD30 staining of the lymphoma cells in the cervical lymph node biopsy. (c) Peripheral blood smear showing eosinophilia with partial degranulation and hypersegmentation. (d) Bone marrow core biopsy showing hypercellular marrow with markedly increased eosinophil precursors and eosinophils. (e) Bone marrow aspirate showing increased eosinophilic precursors, eosinophils with morphology similar to peripheral blood eosinophils and background dyserythropoiesis.

**Figure 2:**
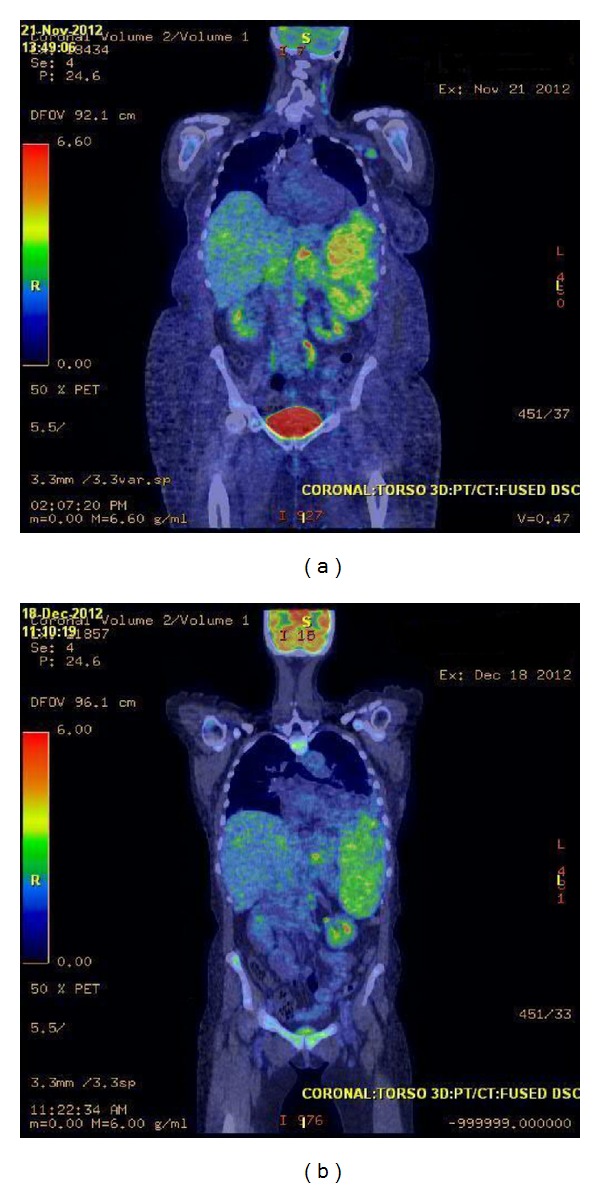
(a) PET/CT scan prior to chemotherapy. (b) PET/CT scan after 2 cycles of chemotherapy.

A 51-year-old female was referred to our medical center for evaluation of peripheral eosinophilia accompanied by a two-month history of progressive shortness of breath, fatigue, weight loss, and fevers. Physical examination demonstrated diminished breath sounds in the right lung along with bilateral cervical lymphadenopathy and hepatosplenomegaly. Further evaluation uncovered a right-sided pleural effusion which was drained and revealed an exudative fluid containing CD30^+^ lymphoma cells. She subsequently underwent an excisional biopsy of her cervical lymph node confirming a CD30^+^, CD20^−^, and ALK^−^ large cell lymphoma, favoring B-cell lineage (Figures 1(a) and 1(b)), with coexpression of CD79a, PAX-5, and MUM-1. A complete blood count during this presentation revealed a white blood cell count of 178 × 10^9^/L, platelets of 56 × 10^9^/L, and hemoglobin of 9.3 grams/dL. A manual differential of the white blood cells showed 4% neutrophils, 90% eosinophils (absolute count of 160 × 10^9^/L), 1% monocytes, 1% basophils, and 4% lymphocytes. She had already been initiated on hydroxyurea at a dose of 3000 mg per day several weeks prior to our evaluation to lower the eosinophil count with no success. Her serum lactate dehydrogenase was elevated at 2,450 Units/L, and a PET/CT scan showed extensive FDG avidity in the spleen, liver, and lymph nodes around the cervical, axillary, and mesenteric regions (Figure 2(a)). A detailed review of her travel history, medication list, past medical history, and previous allergy/atopy reactions were unrevealing for a secondary cause of this eosinophilia.

A peripheral blood smear confirmed the marked eosinophilia with partial degranulation and hypersegmentation (Figure 1(c)). Bone marrow examination demonstrated a hypercellular marrow (90%) with increased eosinophilic precursors and eosinophils (Figure 1(d)). There is background dyserythropoiesis and dysmegakaryopoiesis (Figure 1(e)). There was no evidence of increase in blasts or involvement by lymphoma. Conventional G-banding cytogenetics revealed a complex karyotype in 2 of 18 metaphases, 47-51,XX, dup(1)(q12q42), inv(1)(p36.3q42),add(2)(p23),del(6)(p21.3)[1],-7[1],+9[1],+11,del(13)(q12q21)[1],+18, +add(19)(p13.1),add(22)(q13), +mar[cp2]/46,XX [16], including a 1q duplication, and monosomy 7, which are recurrent findings in myeloid malignancies. Translocation involving FGFR1on chromosome 8p11 was not identified by cytogenetic testing. Interphase fluorescent in situ hybridization (FISH) testing was negative for 5q33, 4q12 gene region abnormalities corresponding to *PDGFRB and PDGFRA *gene rearrangements. A normal tryptase immunostaining pattern of the bone marrow biopsy and absence of *KIT Asp816Val* mutation by allele-specific polymerase chain reaction (PCR) ruled out the presence of systemic mastocytosis; moreover the negative *BCR-ABL* fusion by FISH analysis excluded chronic myelogenous leukemia. An aberrant T-cell immunophenotype was not detected by flow cytometry nor was there a clonal T-cell receptor gene rearrangement. In light of the persistent and marked eosinophilia resistant to hydroxyurea therapy, background dyserythropoiesis and dysmegakaryopoiesis, and the complex karyotype revealing a myeloid neoplasm, the patient's eosinophilia was felt to be most consistent with part of the myeloid clone and would therefore be best classified as chronic eosinophilic leukemia, not otherwise specified (CEL-NOS) per the 2008 World Health Organization (WHO) classification of myeloid malignancies [5]. Altogether her clinical picture suggested the presence of two coexistent malignant processes: (1) a lymphoid neoplasm represented by the CD30^+^ non-Hodgkin's lymphoma (NHL) and (2) a myeloid neoplasm, CEL-NOS.

She had substantial liver dysfunction on presentation with a total bilirubin of 8.1 mg/dL and a direct bilirubin of 7.2 mg/dL that was thought to be secondary to diffuse NHL involvement. Her clinical symptoms were secondary to her pleural effusion and hepatosplenomegaly, both of which were considered to be driven by her NHL; thus lymphoma-directed therapy was pursued. With the existing liver dysfunction, traditional anthracycline containing chemotherapy regimens such as CHOP (cyclophosphamide, doxorubicin, vincristine, and prednisone) was unable to be administered safely. She began treatment with high-dose methylprednisolone (one gram twice daily) for 3 days with no response in her clinical symptoms and liver function tests; however, her peripheral eosinophilia did decrease to an absolute count of 20 × 10^9^/L. One cycle of methachloramine (6 mg/m^2^: days 1 and 8) was then administered with marginal improvement of her total bilirubin decreasing to 5.0 mg/dL. Given the lack of substantial disease response, she was treated with ifosfamide (5000 mg/m^2^: day 1) and carboplatin (AUC 5: day 1) that decreased her total bilirubin to 2.0 mg/dL. This treatment was repeated in three weeks upon her blood count recovery after which her total bilirubin normalized to 1.0 mg/dL; repeat PET/CT scan showed a decrease in the previous FDG avid regions consistent with a partial response (Figure 2(b)).

With normalization of her liver function, she was started on the CHOP chemotherapy regimen administered every 3 weeks with plans to complete four additional cycles aiming for a complete response. Her peripheral eosinophilia persisted with an absolute count ranging from 2 to 5 × 10^9^/L during chemotherapy even after her NHL had evidence of significant regression. She was to be considered for an allogeneic stem cell transplant to help eradicate her myeloid clone responsible for her CEL-NOS once she had completed therapy for her NHL. Unfortunately, while into her second cycle of CHOP chemotherapy, she experienced an aggressive CNS relapse of her NHL associated with significant neurologic compromise prompting her family to pursue comfort care measures and the patient passed away a few days later.

## 3. Discussion

CEL-NOS constitutes a rare entity within the WHO 2008 classification [5] defined by unexplained eosinophilia greater than 1.5 × 10^9^/L either with evidence of clonal eosinophilia via abnormal cytogenetics (excluding *BCR-ABL*, *PDGFRA*, *PDGFRB*, or *FGFR1 *rearrangements) or in the presence of greater than 2% or 5% blasts (but less than 20%) in the peripheral blood or bone marrow, respectively. Its incidence is estimated by a report from the surveillance, epidemiology, and end results database at 0.036 per 100,000 person-years although this includes patients with hypereosinophilic syndrome [2]. The differential diagnosis in a patient with peripheral eosinophilia is extensive and common secondary causes need to be ruled out before considering rarer clonal etiologies. Some such causes include parasitic infections, hypersensitivity conditions, drug reactions, collagen-vascular diseases, and pulmonary eosinophilic diseases [6, 7]. These alterative etiologies were excluded to the best of our ability in the evaluation of this patient via history, physical exam, and laboratory testing. It is not uncommon for nonmyeloid malignancies such as T-cell lymphomas [3] and Hodgkin's disease [4] to be associated with secondary eosinophilia due to the production of cytokines such as IL-3, IL-5, and GM-CSF which promote eosinophil differentiation and survival. This was considered in our patient's case given the concurrent diagnosis of a CD30^+^ NHL although a B-cell lineage was favored in this lymphoma. Even though we cannot completely exclude a minor contribution of secondary eosinophilia related to the lymphoma, the overall clinicopathologic features of this patient's eosinophilia best fit the category of CEL-NOS considering the constellation of findings including marked eosinophilia resistant to hydroxyurea at presentation, dampened yet persistent eosinophilia following significant clinical regression of the NHL with intensive chemotherapy (CHOP), background dyserythropoiesis and dysmegakaryopoiesis supporting CEL, and abnormal cytogenetic findings revealing a myeloid clone. The observed eosinophilic dysplasia, although neither specific nor diagnostic of an eosinophilic clone by itself, in the context of the other findings, is in keeping with the diagnosis of a CEL-NOS. Nonetheless, it is still unclear as to which disease entity arose first, the NHL or the CEL-NOS.

Unlike clonal eosinophilic disorders secondary to *PDGFRA* and *PDGFRB* translocations [8], the WHO-defined CEL-NOS entity is not responsive to imatinib mesylate monotherapy. Furthermore, its prognosis is poor as it is characterized by an aggressive clinical course that is usually unresponsive to conventional chemotherapy and frequently transforms to acute leukemia [9]. In a trial consisting of a cohort of ten patients, the median survival was 22 months, five of whom developed acute transformation to leukemia after a median of 20 months from diagnosis [10]. One of the five patients who did not develop AML underwent an allogeneic stem-cell transplant and maintained a long-term remission [10]. The role of transplantation in CEL-NOSis not well established but appears to be the only curative option available [10, 11]. Interferon-alpha can produce hematologic and cytogenetic remissions in CEL patients refractory to other therapies including prednisone and/or hydroxyurea [12, 13]. Finally, even though this patient's eosinophilia seemed to respond to high-dose systemic corticosteroids, these drugs should not be used long term due to side effects such as hypertension, hyperglycemia, osteoporosis, myopathy, cataracts, and glaucoma.

This case highlights the unique presentation of a patient with a CD30^+^ large cell lymphoma with coexisting CEL-NOS. Moreover, it also demonstrates the difficulty in managing such patients with aggressive lymphoma with impaired liver function.
